# Supplementary material for: Mutations in PCYT2 disrupt etherlipid biosynthesis and cause a complex hereditary spastic paraplegia
Source: Brain. 2019 Oct 22;142(11):3382–97. doi: 10.1093/brain/awz291 (PMC6821184; doi:10.1093/brain/awz291)
Supplement: awz291_Supplementary_Data [file awz291_supplementary_data.zip › awz291-Suppl_data/Supplementary_Data4.zip!/Supplementary information 4 - plasma lipidomics.zip!/Plasma lipidomics/Control vs Patient/Statistical plots and data/TG species/Volcano plot_alternative.pdf]

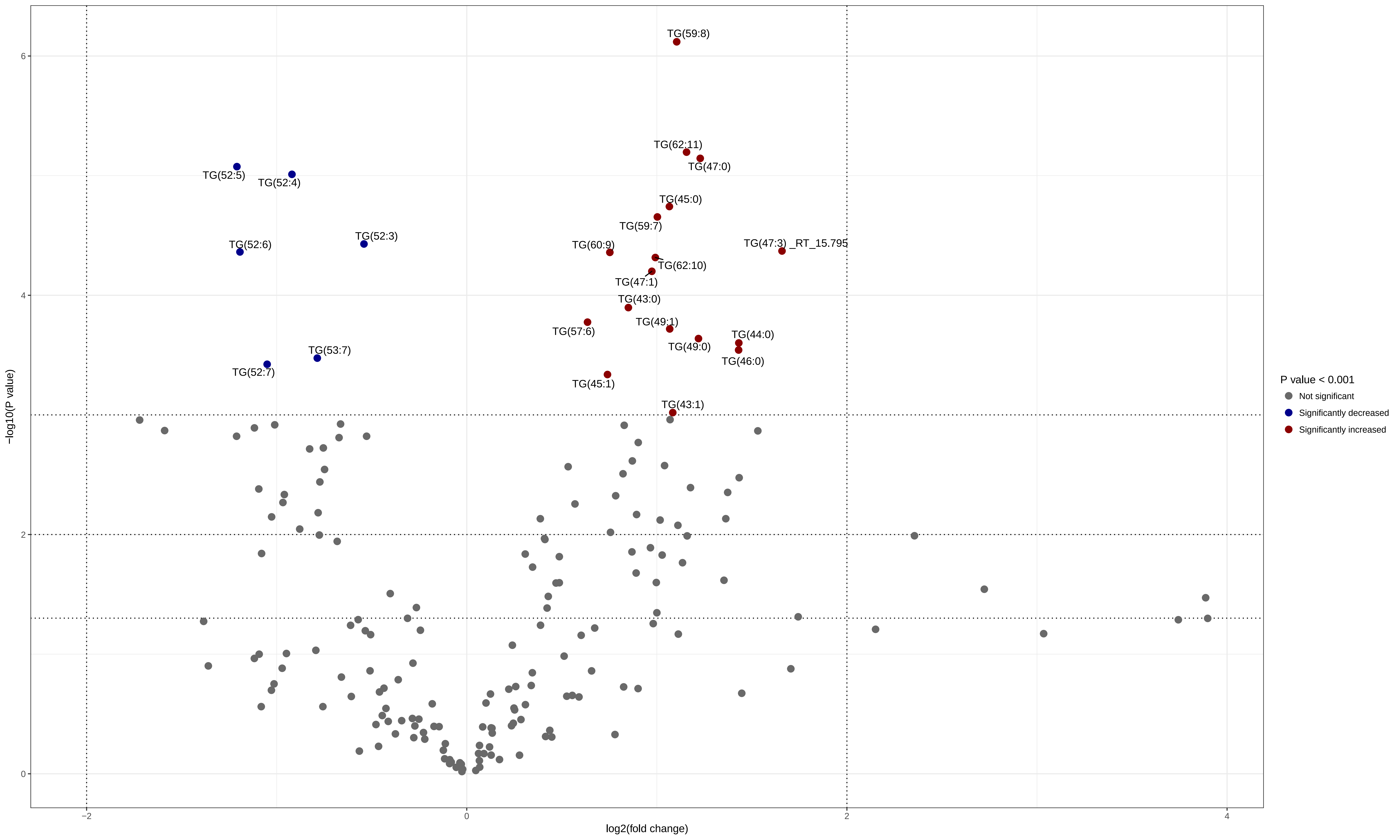

TG(59:8)
TG(62:11)
TG(47:0)
TG(52:5)
TG(52:4)
TG(45:0)
TG(59:7)
TG(52:6)
TG(52:3)
TG(60:9)
TG(47:3)_RT_15.795
TG(62:10)
TG(47:1)
TG(43:0)
TG(57:6)
TG(49:1)
TG(49:0)
TG(44:0)
TG(46:0)
TG(53:7)
TG(52:7)
TG(45:1)
TG(43:1)
−log10(P value)
log2(fold change)
P value < 0.001
Not significant
Significantly decreased
Significantly increased
